# Supplementary material for: A Numerical Investigation of Enhancing Microfluidic Heterogeneous Immunoassay on Bipolar Electrodes Driven by Induced-Charge Electroosmosis in Rotating Electric Fields
Source: Micromachines (Basel). 2020 Jul 30;11(8):739. doi: 10.3390/mi11080739 (PMC7463963; doi:10.3390/mi11080739)
Supplement: Supplementary file 1 [file micromachines-11-00739-s001.pdf]

A numerical investigation of enhancing microfluidic heterogeneous immunoassay on bipolar electrodes driven by induced-charge electroosmosis in rotating electric fields—Supplementary Information

Zhenyou Ge [1], Hui Yan [1,*], Weiyu Liu [3,*], Chunlei Song [1], Rui Xue [1] and Yukun Ren [1,2]

[1]School of Mechatronics Engineering, Harbin Institute of Technology, West Da-zhi Street 92, Harbin 150001, China

[2]State Key Laboratory of Robotics and System, Harbin Institute of Technology, West Da-Zhi Street 92, Harbin, Heilongjiang, PR China 150001

[3]School of Electronics and Control Engineering, Chang'an University, Middle-Section of Nan'er Huan Road, Xi'an 710064, China

*Correspondence: yanhui@hit.edu.cn (H.Y.), Fax: +86-0451-86402658, Phone: +86-0451-86418028; liuweiyu@chd.edu.cn (W.L.), Fax: +86-029-82334555, Phone: +86-029-82334543

SI Appendix 1: Numerical modeling

A commercial software package, Comsol Multiphysics 5.5, is employed to obtain the complex electric field, ICEO vortex flow field, spatial-temporal delivery of antigen, and surface reaction by fully coupled finite-element simulation. Since ROT-ICEO involves out-of-plane effect in essence, full scaled simulation modeling is conducted with a 3D computational domain (Fig.1 for the static case, and Fig.2 for the dynamic condition).

Firstly, we computed the AC potential within the fluid domain by using the Laplace equation for the complex voltage Eq.(1). All the electrodes are subjected to the Robin-type RC charging boundary condition Eq.(2), to describe the phenomenon of electrode polarization. Fixed potential phasor A, jA, -A, and -jA, are imposed to the four discrete DE metal strip along the clockwise direction in sequence with a 90° phase shift between neighboring electrodes, while the square FE in the field center floats in potential. Insulation condition was imposed to all the channel walls due to the negligibly small polarizability of PDMS material.

Secondly, as for the mechanical problem governed by Eq.(4)(5), all the electrodes are subjected to the expression of ICEO slipping velocity Eq.(3), while we assume a no slip wall boundary condition on other insulating channel walls in the presence of a viscous boundary layer. The amplitude, frequency, and phase sequence of the AC voltage signal, as well as the discrete electrode arrangement, all exert an influence on the ICEO vortex flow profile on top of the FE. As a consequence, improvement of the bound antigen would depend sensitively on the selection of the above boundary conditions.

Thirdly, the mass conservation of antigen concentration in the regime of dilute species was solved by using Eq.(6)(7) inside the channel. Flux swap condition $\mathrm{D}\,\partial c/\partial y = \partial B/\partial t$ is imposed on the ideally polarizable surfaces of all the FE, indicating a dynamic equilibrium between the increase of the bound antigen concentration and dissipation of the target analyte from the surrounding liquid medium during the binding reaction. Besides, normal component of the total mass flux vanishes at all other non-functionalized surfaces. The surface concentration of bound antigen at t=0s was B(t=0)=0mol/m^2, signifying no binding reaction at the early stage, while the background concentration of freely suspended

antigen in the liquid bulk was c_0=0.1nM. At last, the physical problem was enclosed by applying the surface reaction equation Eq.(8) at the functionalized interface.

The various parameters adopted the following reference values in the numerical simulation: liquid conductivity σ=0.001S/m, water dielectric permittivity $\varepsilon=80\varepsilon_0$, solution dynamic viscosity η=0.001Pa·s, AC voltage amplitude V_0=4V, Debye screening length λ_D=37.6nm, surface concentration of immobilized antibody $R_T=3.3\times10^{-8}$mol/m^2, the initial volumetric concentration of suspended antigen c0=0.1nM, and the diffusion coefficient of target antigen D=10^{-11}m^2/s. The association and disassociation rate constant for the specific binding reaction are k_{on}=1000m^3/(s·mol) and k_{off}=0.001/s, respectively. The width of FE equals W_F=100μm. The length and depth of the microfluidic chamber are L_C=1mm and H_C=200μm, respectively. This standard parametric space results in a dimensionless Damkohler number Da= $k_{on}R_TH_C/D$=660. The RC time constant for electrochemical polarization of the DE array and FE are $\tau_{RC}^{FE}=RC_D/\sigma(1+\delta)$ =1.99×10-4s and $\tau_{RC}^{DE}=L_CC_D/\sigma(1+\delta)$ =1.84×10-3s, respectively. Correspondingly, the characteristic RC charging frequency for ACEO on the DE array and ICEO on the FE is $f_{RC}^{FE}=\sigma(1+\delta)/2\pi RC_D$ =800Hz and $f_{RC}^{FE}=\sigma(1+\delta)/2\pi L_CC_D$ =86Hz, respectively, with the FE's macroscopic polarization length scale R=0.108W_F. A stationary solver is employed to calculate the steady-state electrostatic potential and time-averaged ROT-ICEO fluid motion in sequence. Then a transient solver is adopted to compute both the analyte transport in the bulk and the binding reaction at the functionalized surface in a coupled way considering their time-dependent nature.

SI Appendix 2: A comparative study of various ICEO convection modes

It is necessary to identify the various convection modes of ICEO when the peripheral DE array is powered by different voltage sequence combinations. Three powering schemes are considered in current analysis, including (i) $V_1=V_0$cos(wt), V_2= V_0cos(ωt+90°), V_3= V_0cos(ωt+180°), $V_4=V_0$cos(ωt+270°) with neighboring DE phase shifted by 90°, (ii) V_1= V_0cos(ωt), $V_2=V_0$cos(ωt), $V_3=V_0$cos(ωt+180°), $V_4=V_0$cos(ωt+180°) with every two

electrodes phase shifted by 180°, (iii) $V_1=V_0\cos(\omega t)$, $V_2=V_0\cos(\omega t+180°)$, $V_3=V_0\cos(\omega t)$, $V_4=V_0\cos(\omega t+180°)$ with adjacent DE oppositely polarized. (Table 1)

First and foremost, we focus attention on case (i), namely, the situation of ROT-ICEO. As shown in the bi-dimensional phase diagram of ROT-ICEO (Fig.3), at every time instant of a particular one AC cycle, the induced counterions on the surface of central FE always manifest as a bipolar charge pattern, and the net charge profile rotates synchronously with the background rotating field in the low-frequency limit. The dot product of the induced space charge and the applied rotating field gives rise to the ROT-ICEO slipping flow on top of the blocking FE, which quickly switches between two mutually orthogonal convection modes (Fig.3(a)(c) and Fig.3(b)(d)) reminiscent of the typical ICEO pattern in a standing-wave AC forcing, resulting in conceivably a radially converging flow profile tending to transport fluidic samples from surrounding liquid medium to the center of the blocking FE after time-averaging, in accordance with the simulation result in Fig.S4(a). As for case (ii), since the voltage phase was sequentially shifted by 180° for every two electrodes (Fig.S1(a)(b)), the externally-applied electric field lines under the excitation of a such voltage sequence are inclined with respect to the central FE, as shown in Fig.S1(a), and just alternates in direction with time elapses (Fig.S1(b)). Even so, the converging ICEO flow pattern parallel to the field lines remains unchanged in slipping direction (Fig.S1(a)(b)), in that both the induced charge and applied electric field reverse direction at a fixed time interval equaling half of the harmonic cycle, which is in qualitative agreement with the simulation result in Fig.S5(c). In case (iii), though the schematic diagram indicates the slipping flow streams inward from all directions on the ideally polarizable surface of the central FE (Fig.S1(c)(d)), the ICEO flow velocity is too weak to play the role of strengthening the binding reaction, as predicted by the simulation result in Fig.S6(c) as well.

All the above calculations of ICEO flow field are obtained at a voltage amplitude of $V_0=4V$ and respective optimum field frequency. According to the calculation results, the ICEO flow velocity shows strong frequency-dependence for all the three convection modes (Fig.S2(b)), and the same also holds true for their ACEO counterpart on the DE array (Fig.S2(a)). Both the ICEO and ACEO flow fields reach a single relaxation peak at an

intermediate field frequency, regardless of the specific power supply modes (Fig.S2(a)(b)). In DC limit, since all of the applied voltage drops across the IDL on the DE array due to complete Debye screening, there is no tangential field gradient acting on the induced charge within the Debye layer, resulting in negligibly small ACEO slipping on the DE array and ICEO slipping on the central FE. At higher field frequencies, there is insufficient time for the counterionic charges to become accumulated effectively within the thin IDL due to the occurrence of electrochemical ion relaxation, giving rise to extremely slow ICEO fluid motion once again. As a result, both ACEO and ICEO are maximized in an intermediate frequency range, as defined by the inverse RC time constant for double-layer capacitive charging on the FE $f_{RC}^{DE} = \sigma(1+\delta)/2\pi L_C C_D$ =86Hz and that on the DE $f_{RC}^{FE} = \sigma(1+\delta)/2\pi RC_D$ =800Hz serving as the low and high frequency threshold, respectively. As shown in Fig.S2(a), ACEO for both (i) and (ii) convection modes reaches a single relaxation peak at f_{ideal}=200Hz, while case (i) possesses a much broader force plateau than case (ii). The ideal driving frequency of ACEO for case (iii) attains 300Hz, being higher than that for case (i) and (ii), and its maximum ACEO flow velocity is highest among the three convection modes.

As for ICEO, the opposite has happened. The (iii) powering scheme results in the weakest ICEO fluid motion than other two convection modes, with the typical ICEO flow velocity merely on the order of O(1)μm/s (Fig.S2(b)), albeit its ACEO counterpart is strongest (Fig.S2(a)). ICEO for case (i) and (ii) exhibits an identical frequency-dependent variation trend with a slightly larger flow speed of case (ii) than that of case (i). Even so, the convection mode of ROT-ICEO driven by case (i) is supposed to be able to engender better improvement of the antigen-antibody binding reaction due to its more favorable slip profile that the liquid medium is sucked at the electrode edge from all outer directions and is then ejected upward at the center of the electrode surface whatever the signal frequency is (Fig.S4(a-d)), in stark contrast with the linear slipping fluid motion for case (ii) (Fig.S5(c)) and the vanishing ICEO flow field for case (iii) (Fig.S6(c)). In addition, in case (i), wherein a rotating electric field emits from the peripheral DE array, ACEO has a net counterclockwise rotating flow component in the direction of the rotating electric field (Fig.S3(a)(d)). This large whirlpool induced in the field center dominates the global

electrokinetic flow behavior at low frequencies, (Fig.S3(a)(d)(b)(e)), and diminishes as the field frequency further increases (Fig.S3(c)(f)). In this way, it is more beneficial to employ the convergent ICEO slipping pattern (Fig.S4) on top of the central FE and global ACEO whirlpool in counterclockwise rotating direction enabled by case (i) for accelerating the binding rate between antigen and antibody on application of a low-frequency rotating electric field to the peripheral DE array, than other two powering schemes including case (ii) and (iii).

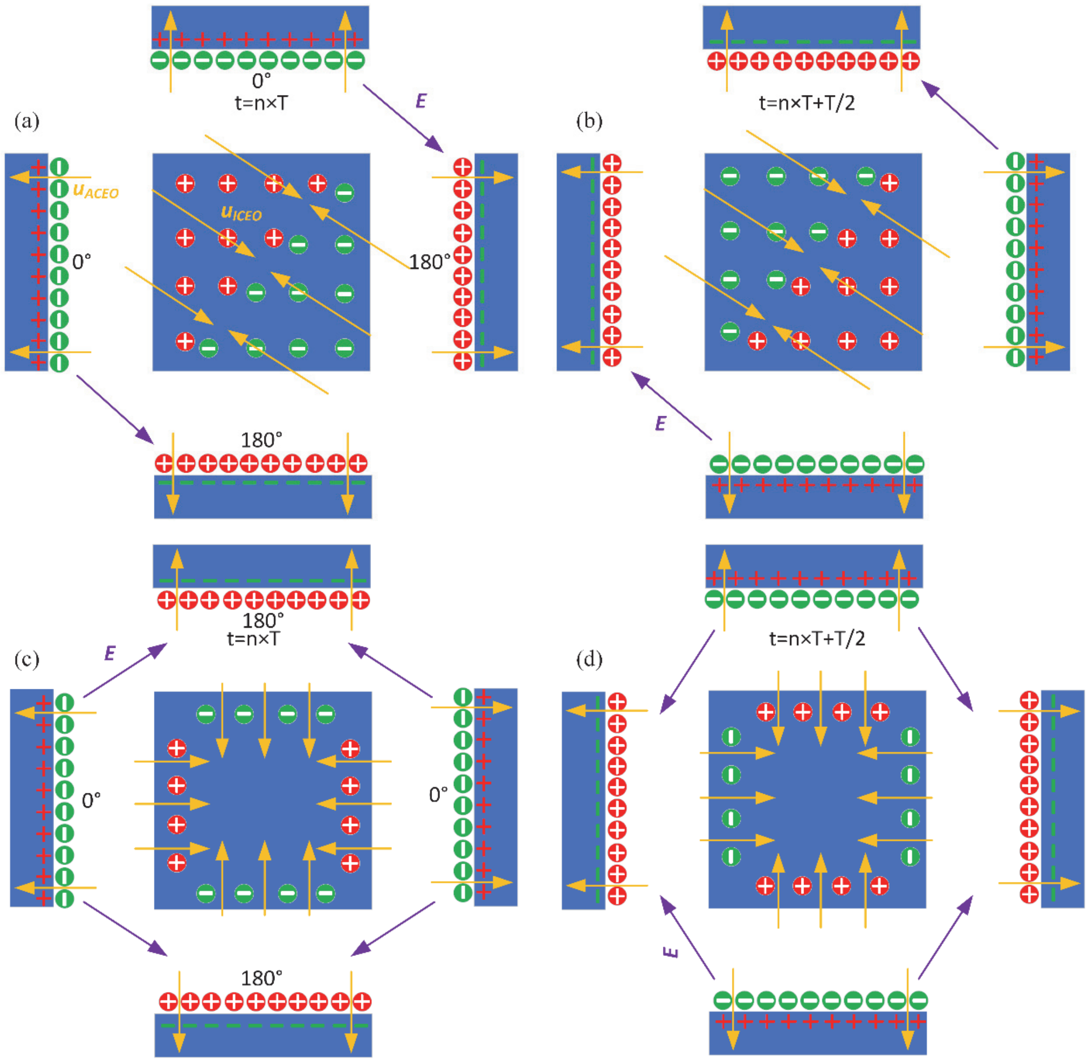

Figure S1. (a)-(b) Time sequence of the phase diagram for case (ii) with every two DE phase shifted by 180°, (a) at t=nT, and (b) at t=nT+T/2. (c)-(d) Time-sequence of the phase diagram for case (iii) with adjacent DE being oppositely polarized, (a) at t=nT, and (b) at t=nT+T/2.

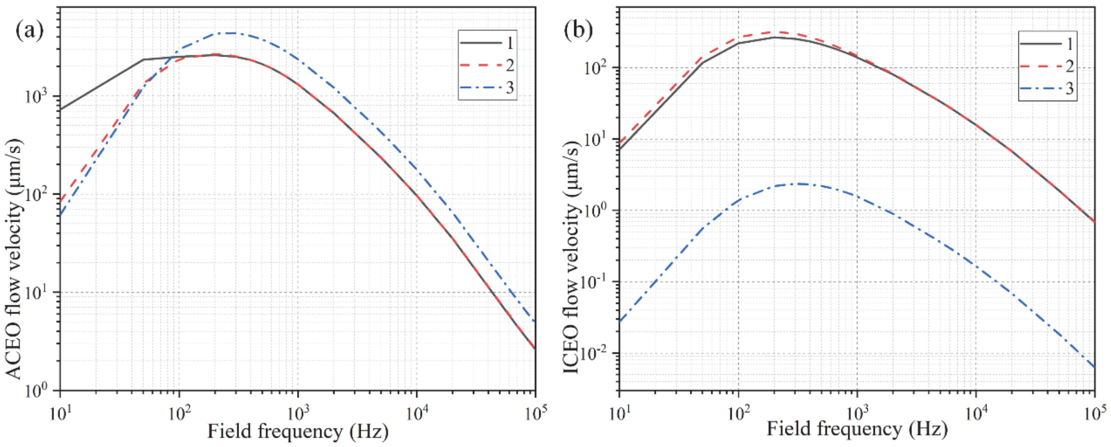

Figure S2. A comparison of the frequency-dependence of electrokinetic flow velocity between different convection modes ((i), (ii) and (iii)) at a given voltage amplitude of V_o=4V. (a) ACEO flow velocity on top of DE array as a function of the signal frequency. (b) ICEO flow speed on top of the central FE as a functional of the electric field frequency.

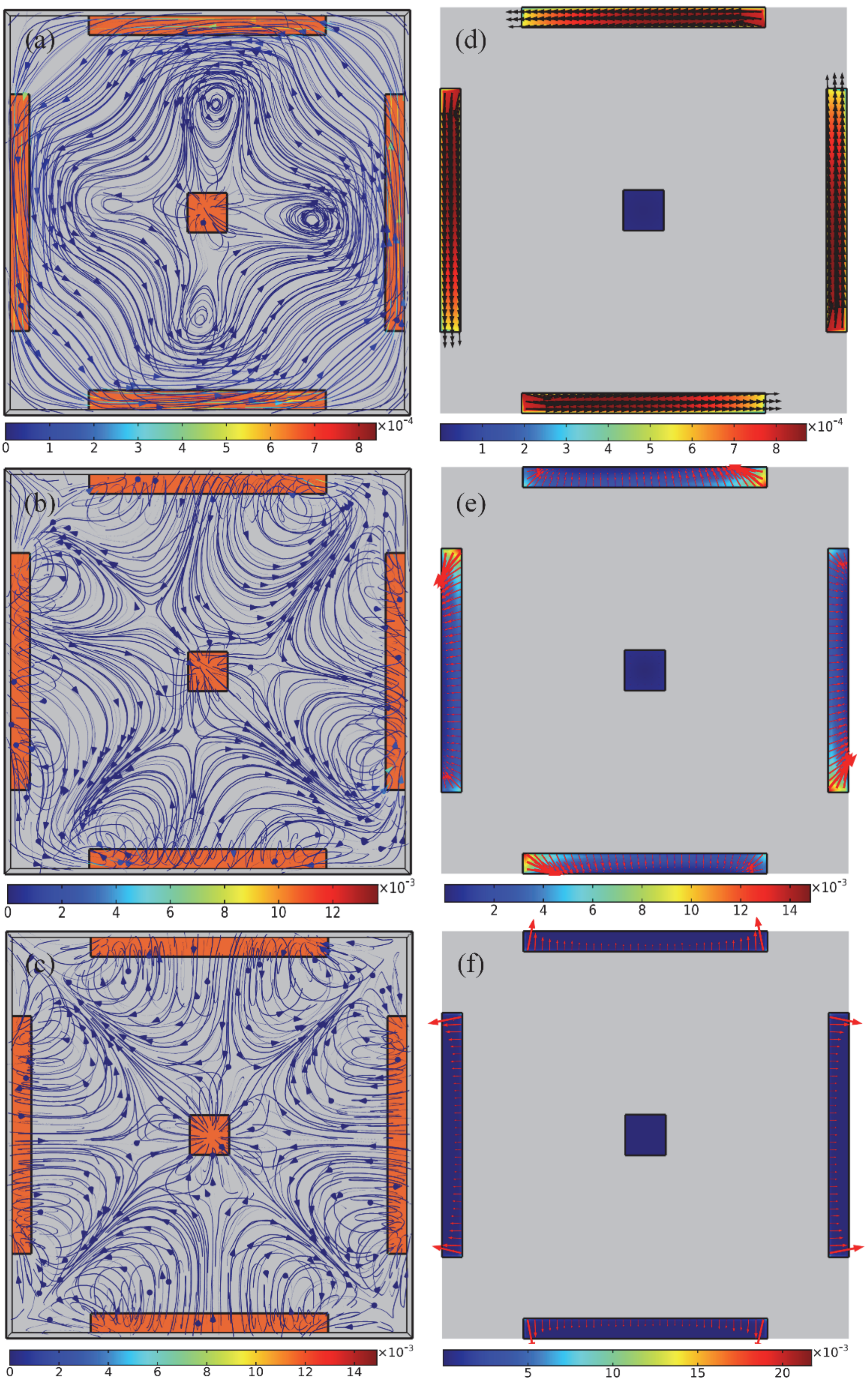

Figure S3. Simulation result of ROT-ICEO flow field inside the static fluidic chamber for case (i) under a given voltage amplitude V_0=4V. (a)-(c) A top view of the tri-dimensional streamline plot of combined ACEO and ICEO flow field at distinct frequency points, (a) f=10Hz, (b) f=200Hz, and (c) f=5000Hz. (d)-(f) An arrow and surface plot of ICEO flow field right on the ideally polarizable surfaces of the electrode array under different

excitation frequencies, (d) f=10Hz, (e) f=200Hz, and (f) f=5000Hz. (unit: m/s)

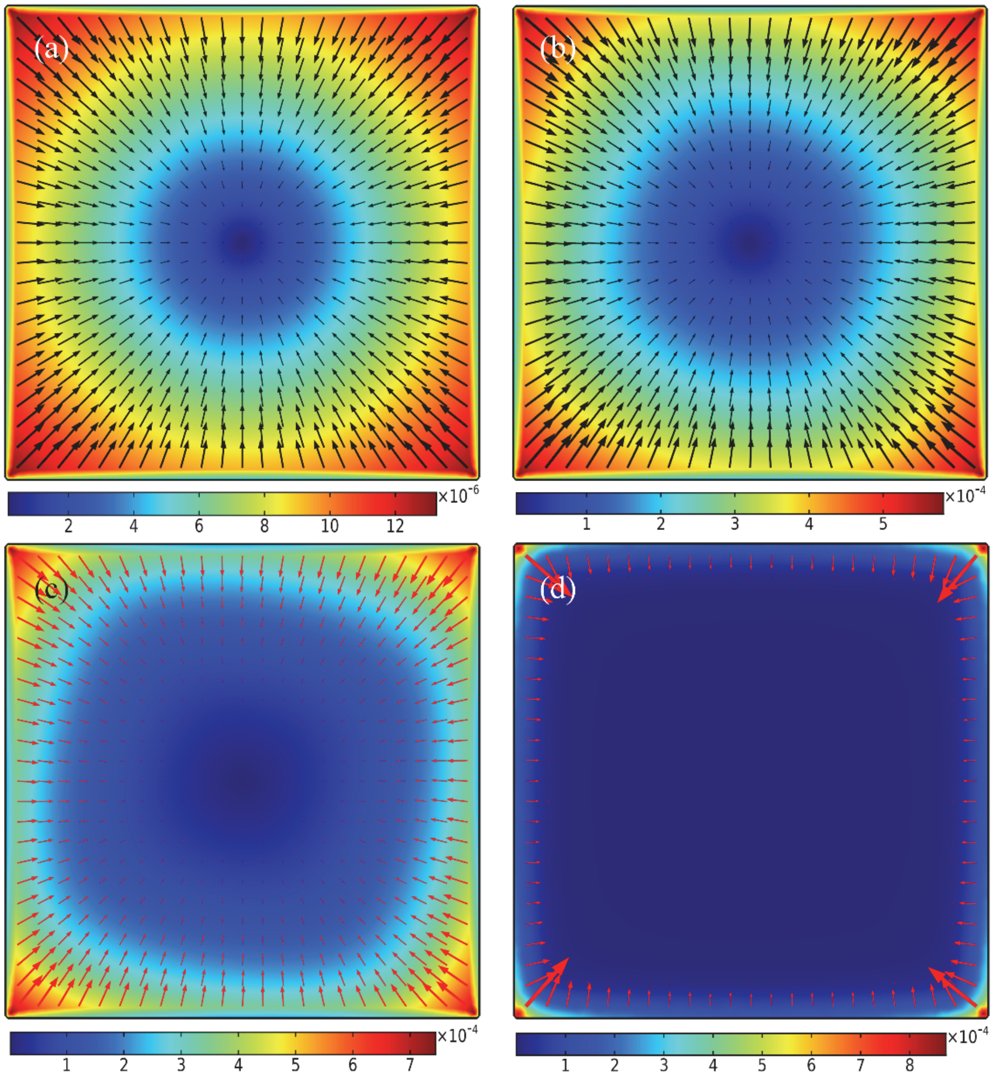

Figure S4. A surface and arrow plot of ROT-ICEO slipping fluid motion on the central FE for case (i) at distinct signal frequencies: (a) f=10Hz; (b) f=200Hz; (c) f=500Hz; d) f=5000Hz. (unit: m/s)

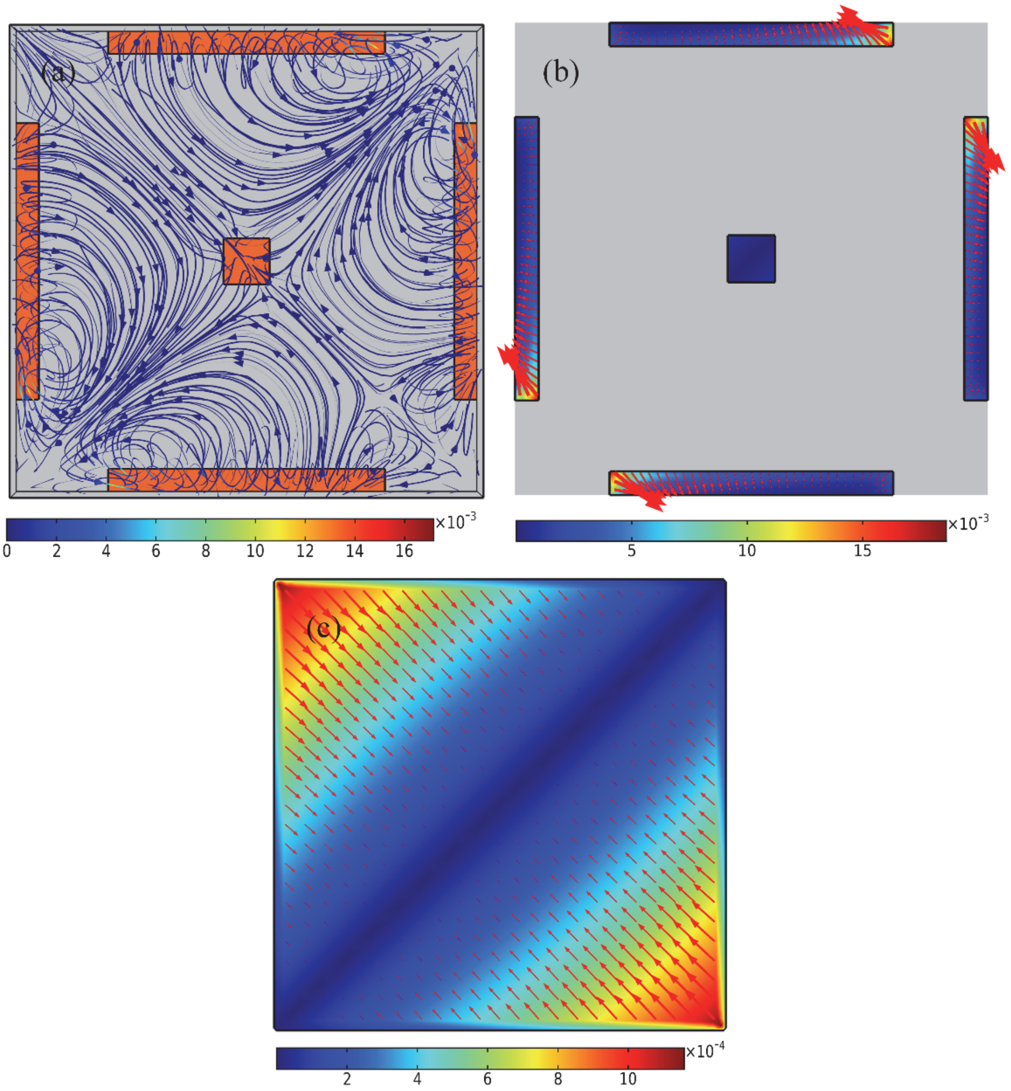

Figure 8. B 情况

Figure S5. Simulation result of nonlinear electroosmotic flow field within the microchamber for case (ii) at f=200Hz and V_0=4V. (a) A top view of tri-dimensional streamline plot of the combined ACEO and ICEO vortex flow field within the fluidic chamber. (b) A surface and arrow plot of ACEO slipping fluid motion on the electrode array. (c) A surface and arrow plot of ICEO slipping fluid motion on the central FE. (unit: m/s)

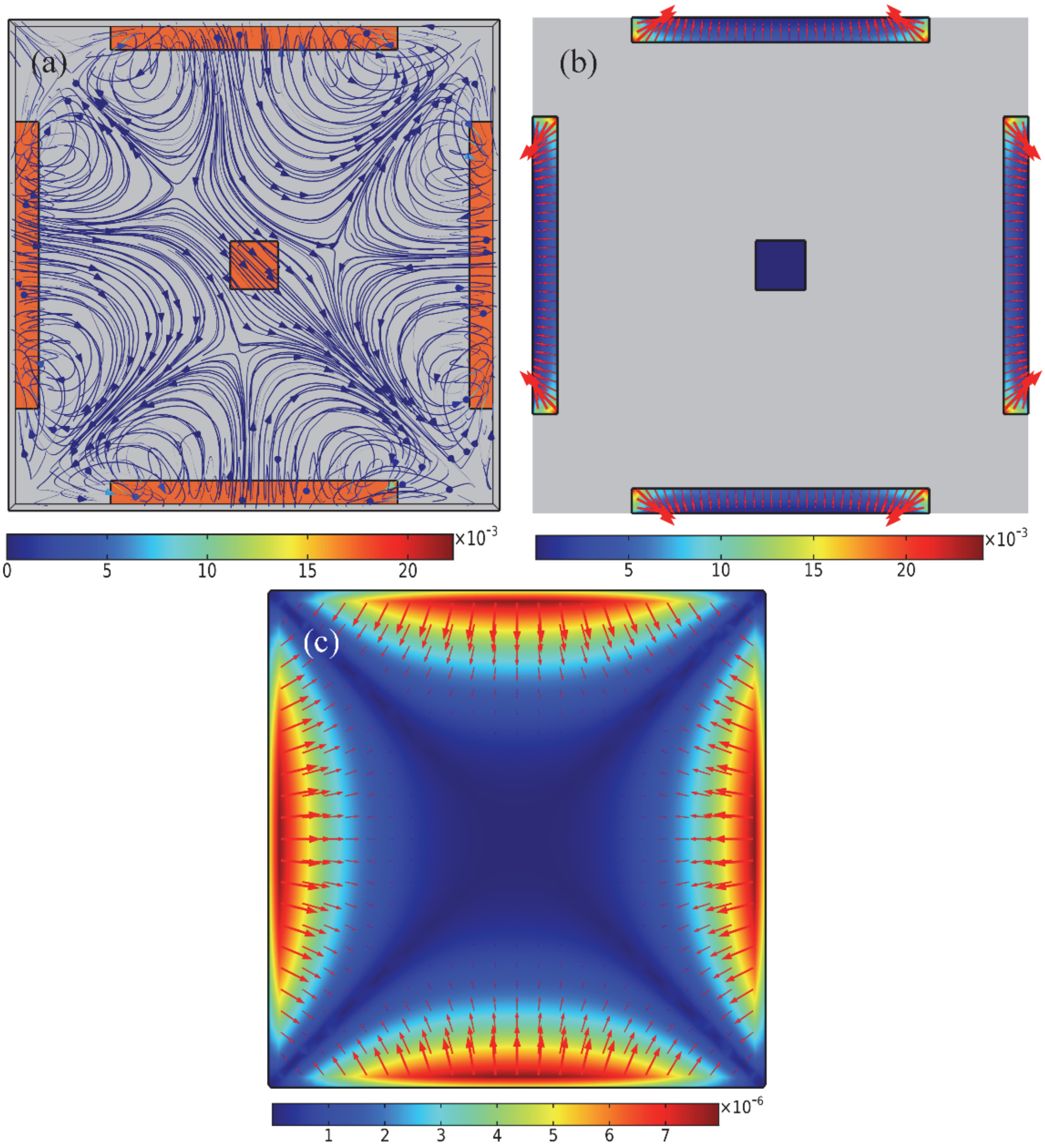

Figure S6. Simulation result of nonlinear electroosmotic flow field within the microchamber for case (iii) at f=300Hz and V_0=4V. (a) A top view of tri-dimensional streamline plot of the combined ACEO and ICEO vortex flow field within the fluidic chamber. (b) A surface and arrow plot of ACEO slipping fluid motion on the electrode array. (c) A surface and arrow plot of ICEO slipping fluid motion on the central FE. (unit: m/s)
